# Diagnostic Validity of Patient-Reported History for Shoulder Pathology

**DOI:** 10.1055/s-0037-1601878

**Published:** 2017-04-24

**Authors:** Lyndsay E. Somerville, Kevin Willits, Andrew M. Johnson, Robert Litchfield, Marie-Eve LeBel, Jaydeep Moro, Dianne Bryant

**Affiliations:** 1 Department of Surgery, Schulich School of Medicine & Dentistry, The University of Western Ontario, London, Ontario, Canada; 2 School of Health Studies, Faculty of Health Sciences, The University of Western Ontario, London, Ontario, Canada; 3 Division of Orthopaedic Surgery, Department of Surgery, McMaster University, Hamilton, Ontario, Canada; 4 Department of Clinical Epidemiology and Biostatistics, Faculty of Health Sciences, McMaster University, Hamilton, Ontario, Canada

**Keywords:** shoulder, diagnosis, history

## Abstract

**Objective** The purpose of this article is to determine whether patient-reported history items are predictive of shoulder pathology and have the potential for use in triaging patients with shoulder pathology to orthopaedic outpatient clinics.

**Setting** It is set at two tertiary orthopaedic clinics.

**Patients** All new patients reporting pain and/or disability of the shoulder joint were prospectively recruited. A total of 193 patients were enrolled, 15 of whom withdrew, leaving 178 patients composing the study sample.

**Design** Patients completed a questionnaire on the history of their pathology, then the surgeon took a thorough history indicating the most likely diagnosis. The clinician then performed appropriate physical examination. Arthroscopy was the reference standard for those undergoing surgery and magnetic resonance imaging (MRI) with arthrogram for all others. We calculated the sensitivity, specificity, and likelihood ratios (LRs) of history items alone and in combination. We used the LRs to generate a clinical decision algorithm.

**Main Outcome Measures** Diagnosis was determined through arthroscopy or MRI arthrogram. Reporting was standardized to ensure review of all structures.

**Results** The physical examination and history agreed in 75% of cases. Of those that did not agree, the physical examination misdirected the diagnosis in 47% of our cases. In particular, history items were strong predictors of anterior and posterior instability and subscapularis tears and were combined in a tool to be utilized for screening patients.

**Conclusion** The patient-reported history items were effective for diagnosing shoulder pathology and should be considered for use in a triaging instrument.

Musculoskeletal disorders are the most prevalent chronic health condition in Canada, being both the leading cause of disability and cause the greatest use of health care resources in Canada. 1 Shoulder complaints are the third most common musculoskeletal problem in the general population, second to knee referrals to orthopaedic surgery or primary care sports medicine clinics. 2 Shoulder pain and disability pose a challenge for physicians owing to the numerous etiologies and the potential for multiple disorders existing in the same patient. A thorough history and clinical evaluation of the entire shoulder girdle, along with clinical tests and imaging may be necessary to make a diagnosis. More invasive tests, including magnetic resonance imaging (MRI) and arthroscopic exam, are often felt necessary, as a clinical evaluation alone can frequently lead to misdiagnoses.

Although most physicians rely on these modalities to arrive at a definitive diagnosis, patient history may be sufficient to predict pathologies associated with the shoulder. Over a half century ago, Platt claimed that in most general medical cases, a diagnosis can be made with a history alone. 3 Hampton et al 4 evaluated the importance of the medical history in the diagnosis of general medical outpatients and found that in 83% of their patients, the diagnosis following the history agreed with the final diagnosis. Similarly, Peterson et al 5 found that the history led to a correct diagnosis in 76% of their general medical outpatients. Although this phenomenon has been demonstrated in many patient populations, few studies have evaluated the accuracy of the history as a diagnostic test for shoulder pathology.

Litaker et al 6 demonstrated that age older than or equal to 65 years, and night pain, were the most predictive of rotator cuff tears. Holtby and Razmjou 7 found that 76% of their patients referred for surgery had night pain. Michener et al 8 examined history of trauma, sudden onset of pain, and history of popping, clicking, or catching, and demonstrated that none of these items had diagnostic utility for superior labrum anterior to posterior (SLAP) lesions.

Primary care physicians often misdirect referrals of musculoskeletal conditions to orthopaedic surgeons when nonsurgical intervention is most appropriate. 9 This reduces the efficiency of these services and can potentially affect quality of care. Thus, having a tool to assist with the practice of triage can streamline the care of patients. To this end, Stiell et al 10 developed a clinical decision rule, the Ottawa ankle rules, to guide the assessment of ankle injuries. The Ottawa ankle rules provide a high level of diagnostic confidence and has reduced the number of radiographs ordered by emergency departments. 11 12 Applying the same principle to the shoulder population could reduce the number of patients being referred for further diagnostic tests, thus, improving the efficiency of these services for others.

The purpose of this article is to determine whether patient-reported history items are predictive of shoulder pathology. We will assess whether a clinical decision rule can be developed that could effectively triage patients with shoulder pathology to orthopaedic outpatient clinics.

## Methods

### Patient Population

Using a consecutive sampling strategy, we recruited all participants presenting for their first consultation for shoulder pain or disability between May 2007 and November 2008, within two tertiary care centers that specialize in orthopaedics. We excluded patients with adhesive capsulitis or glenohumeral arthritis. A total of 193 patients participated in this study. All patients gave informed consent, and the study was approved by each center's Research Ethics Board.

### Identification of History Items

We conducted a review of the diagnostic literature for shoulder pathology to identify common items used in a typical clinician history. A list of items was compiled for the most common pathology (rotator cuff pathology, labral pathology, acromioclavicular abnormalities, and instability) and circulated to expert orthopaedic surgeons with a specialty in shoulder disorders for review. In a round-table discussion, each item was reviewed individually by the clinicians and they selected whether to include or exclude the item. Any discrepancies were re-examined until a consensus was reached.

### Clinical Examination Testing

Prior to seeing the clinician, patients completed a detailed questionnaire asking questions in regard to their referred painful/disabled shoulder, which included the items identified by clinicians. These elicited demographic information, symptoms, mechanism of injury, and history of their disease. The clinician was not provided with the completed questionnaire. Instead, the clinician took the patient's history as usual. Following the history, the clinician recorded their primary diagnosis and any secondary diagnoses, then rated their confidence with each diagnosis on a visual analog scale (VAS) ranging from 0 to 100% confidence. The clinician then performed the physical examination maneuvers for any disease suspected to contribute to the patient's symptoms. The clinician was then asked (again) to indicate their primary and any secondary diagnoses and to indicate their confidence in these diagnoses. The results of the physical examination maneuvers are reported elsewhere. 13 14

### Reference Standard

Arthroscopic examination and MRI arthrogram were the main reference standards. We developed a standardized arthroscopic examination and reporting protocol to minimize differences between surgeons in diagnoses due to variations in methods of examination. The clinicians were to look specifically at the subacromial space, rotator cuff tendons, glenoid labrum, acromioclavicular joint, biceps tendon, and cartilage.

Although the majority of patients went on to have surgery, some did not require surgery, or opted out of recommended surgery. These patients underwent a standardized MRI arthrogram as the reference standard. Since the literature has shown that MRI alone is not as accurate for diagnosing SLAP tears, with reported sensitivities for MRI ranging from 43 to 75%, 15 16 17 18 19 and specificities between 58 and 70%, 15 18 19 we included the arthrogram. There is good evidence to suggest that MRI arthrogram is a comparable reference standard to arthroscopy. MRI arthrogram has been shown to be highly sensitive (100 and 82%) and specific (88 and 100%) for detecting SLAP injuries. 20 21

### Plan for Statistical Analysis

Sensitivity and specificity were calculated for each history item including 95% confidence intervals. These values were used to calculate positive and negative likelihood ratios (LRs). LRs greater than 1 increase the probability that the test result is associated with the disease, whereas LRs less than 1 indicate that the test result is associated with the absence of disease.

We calculated the proportion of diagnoses that agreed following the patient history and the physical examination. Among those that agreed, we calculated the proportion that was accurate according to the gold standard. For these patients, we also calculated the change in confidence in the diagnosis following the physical examination. For those patients in whom a discrepancy was noted between the primary diagnosis after the physical examination and the primary diagnosis following the history, we determined the proportion of primary diagnoses that were switched with the secondary diagnosis after the physical examination, and the proportion of primary diagnosis that changed entirely following the physical examination. Of these cases, we calculated the proportion of diagnosis that the history identified correctly and that the physical examination identified correctly according to the gold standard.

We used the LRs to generate a clinical decision rule. The item with the highest LR was selected as the first question in the decision algorithm. All patients who answered “yes” to this question were removed from subsequent analyses, and the measurement properties were recalculated with the new sample. This process was repeated until the remaining history items produced LRs that would not change the clinician's impression of the probability of the target disorder (i.e., the LR was less than 2).

For any disease in which the history items would not change the clinicians impression (LR less than 2), we calculated the prevalence of disease at that step in the algorithm and used this value as the pretest probability. We calculated the 95% confidence interval around this probability. Using the literature on the diagnostic validity of MRI arthrogram, we calculated the LR for MRI arthrogram for any disease that the history items could not diagnose. Using the pretest probability and LR, we calculated the posttest probability of these disorders if an MRI arthrogram was ordered. This value was calculated for the lower and upper 95% confidence interval of the prevalence.

## Results

The clinicians selected 32 items to be included in the patient history questionnaire. The questionnaire consisted of items for anterior instability, posterior instability, multidirectional instability, SLAP lesions, tendinosis, subscapularis disease, rotator cuff disease, and acromioclavicular abnormalities ( Table 1 ).

**Table 1:** Patient-reported history questionnaire items

Q1: Did you try any new activities in the days preceding the onset of pain? Q2: Do you experience pain when performing overhead activities? Q3: Do you feel pain in your shoulder during rest? Q4: Do you have difficulty lifting objects? Q5: At the time of injury, did you feel a snap/tear in your shoulder? Q6: Did the onset of pain in your shoulder occur after a motor vehicle accident (while wearing a seatbelt)? Q7: Do you have weakness in your shoulder when doing up your seatbelt? Q8: Do you have weakness when throwing an object overhand? Q9: Does your occupation or hobbies require elevation of the arm above the level of the shoulder? Q10: Has your shoulder pain been longstanding (> 6 mo)? Q11: Do you experience pain at night while lying on the injured shoulder? Q12: Does pain at night awaken you from your sleep? Q13: Is the pain worsened by participating in activities where the elbow is level with the shoulder? Q14: Do you have a feeling of clicking, clunking, or grinding with use of your arm overhead? Q15: Do you feel weakness in your shoulder without any pain? Q16: Is the pain in your shoulder worsened by the position of your neck? Q17: Do you have numbness/tingling in your hand? Q18: Does your shoulder pain radiate to your hand? Q19: At the time of injury, did you feel a sudden pull on your arm (e.g., waterskiing, grabbing onto something when falling, sudden pull when losing hold of a heavy object)? Q20: Do you participate regularly in overhead sports (e.g., tennis, baseball, squash, etc.)? Q21: Do you experience a catching, locking, popping, or grinding along with pain in your injured shoulder? Q22: Do you ever experience the feeling of your arm coming out of the socket? Q23: Has your shoulder ever dislocated from its socket? Q24: Does your shoulder feel unstable toward the back of your body? Q25: Did your shoulder become painful after a traumatic event (e.g., motor vehicle accident)? Q26: At the time of injury, was your arm driven backward (e.g., car accident while holding the wheel, taking a hit from the front)? Q27: Are you extremely flexible? Q28: Can you make your shoulder come out? Q29: Does your shoulder come out with daily activities? Q30: Do you experience discomfort while doing weight lifting, push-ups, or dips? Q31: Do you feel like your collar bone moves when raising your arm?

Of the 193 enrolled patients, 15 patients refused to undergo one of the reference standard tests, or canceled their scheduled test; therefore, the remaining 178 patients composed the study sample. There were 127 males and 51 females with an average age of 41.8 (standard deviation = 17.5) years.

The diagnostic validity measures for all of the history items are presented in Table 2 . The majority of questions intended to diagnose rotator cuff disease were highly sensitive, but their LRs suggested that they are not clinically useful. The results were similar for subscapularis tears and SLAP tears, but Question 5 (“At the time of injury, did you feel a snap/tear in your shoulder?”) had a LR approaching three for full-thickness tears of the subscapularis. If the history items for subscapularis were assessed in combination, adding Question 8 (“Do you have weakness when throwing an object overhand?”) improved this LR to over three. The majority of history items for posterior instability had poor diagnostic ability, but Question 26 (“At the time of injury, was your arm driven backward?”) and Question 29 (“Does your shoulder come out with daily activities?”) had LRs over two. All of the items for anterior instability were good indicators of disease, with LRs over three.

**Table 2:** Diagnostic validity measures for patient-reported history items

Item	Sensitivity	95% CI	Specificity	95% CI	Positive LR	Negative LR
Rotator cuff disease a						
Q1						
All disease	13.6	8.0–22.3	80.0	70.6–87.0	0.68	1.08
All tears	12.5	6.7–22.1	80.2	71.6–86.7	0.63	1.09
FT tears	11.1	5.5–21.2	81.2	73.3–87.1	0.59	1.10
Tendinosis	18.8	6.6–43.0	83.3	76.8–88.3	1.13	0.98
Q2						
All disease	95.5	88.9–98.2	13.3	7.8–21.9	1.10	0.34
All tears	97.2	90.4–99.2	13.2	8.0–21.0	1.12	0.21
FT tears	96.4	87.9–99.0	11.5	7.0–18.3	1.09	0.31
Tendinosis	87.5	64.0–96.5	8.6	5.2–14.0	1.45	0.66
Q3						
All disease	86.4	77.7–92.0	34.4	25.5–44.7	1.32	0.40
All tears	88.9	79.6–94.3	33.0	24.8–42.4	1.33	0.34
FT tears	87.5	76.4–93.8	29.5	22.1–38.1	1.24	0.42
Tendinosis	75.0	50.5–89.8	24.1	18.1–31.2	0.99	1.04
Q4						
All disease	83.9	74.8–90.2	28.9	20.5–39.0	1.18	0.56
All tears	84.5	74.4–91.1	27.4	19.8–36.5	1.16	0.57
FT tears	85.5	73.8–92.4	26.2	19.2–34.7	1.16	0.56
Tendinosis	81.3	57.0–93.4	23.0	17.2–30.1	1.06	0.82
Q8						
All disease	89.8	81.7–94.5	23.5	15.8–33.6	1.17	0.44
All tears	90.3	81.3–95.2	21.8	14.9–30.1	1.15	0.45
FT tears	92.6	82.5–97.1	21.0	14.7–29.2	1.17	0.35
Tendinosis	87.5	64.0–96.5	17.2	12.1–23.9	1.06	0.73
Q9						
All disease	74.7	64.7–82.7	20.0	13.0–29.4	0.93	1.26
All tears	77.5	66.5–85.6	22.6	15.7–31.5	1.00	0.99
FT tears	78.2	65.6–87.1	23.0	16.4–31.2	1.02	0.95
Tendinosis	62.5	38.6–81.5	21.1	15.5–28.1	0.79	1.78
Q10						
All disease	87.5	79.0–92.9	17.8	11.3–26.9	1.06	0.70
All tears	86.1	76.3–92.3	16.0	10.3–24.2	1.03	0.87
FT tears	83.9	72.2–91.3	14.8	9.5–22.1	0.99	1.09
Tendinosis	93.8	71.7–98.9	16.1	11.2–22.5	1.12	0.39
Q11						
All disease	92.0	84.3–96.1	26.7	18.6–36.6	1.25	0.30
All tears	94.4	86.4–97.8	25.5	18.1–34.5	1.27	0.22
FT tears	94.6	85.2–98.1	23.0	16.4–31.1	1.23	0.24
Tendinosis	81.3	57.0–93.4	17.4	12.3–24.0	0.98	1.08
Q12						
All disease	79.6	70.0–86.7	53.3	43.1–63.3	1.71	0.38
All tears	80.6	70.0–88.1	49.1	39.7–58.4	1.58	0.40
FT tears	83.9	72.2–91.3	46.7	38.1–55.5	1.56	0.34
Tendinosis	75.0	50.5–89.8	38.3	31.1–46.0	1.22	0.65
Q13						
All disease	88.6	80.3–93.7	18.9	12.1–28.2	1.09	0.60
All tears	90.3	81.3–95.2	18.9	12.6–27.4	1.11	0.52
FT tears	91.1	80.7–96.1	18.0	12.2–25.8	1.11	0.50
Tendinosis	81.3	57.0–93.4	14.8	10.2–21.1	0.95	1.27
Q14						
All disease	69.0	58.6–77.7	26.7	18.6–36.6	0.94	1.16
All tears	70.8	59.5–80.1	28.6	20.8–37.9	0.99	1.02
FT tears	69.6	56.7–80.1	28.1	20.9–36.7	0.97	1.08
Tendinosis	60.0	35.8–80.2	27.8	21.5–35.1	0.83	1.44
Q15						
All disease	56.8	46.4–66.7	33.0	24.0–43.3	0.85	1.31
All tears	56.2	44.8–67.0	34.0	25.6–43.6	0.85	1.29
FT tears	54.6	41.5–67.0	34.7	26.8–43.6	0.84	1.31
Tendinosis	60.0	35.8–80.2	37.9	30.8–45.6	0.97	1.06
Subscapularis tears						
Q5						
All disease	44.7	30.2–60.3	66.2	57.8–73.7	1.32	0.84
All tears	57.9	36.3–76.9	66.5	58.6–73.5	1.73	0.63
FT tears	87.5	52.9–97.8	66.3	58.7–73.1	2.59	0.19
Tendinosis	31.6	15.4–54.0	77.8	72.3–82.5	1.42	0.88
Q6						
All disease	2.4	0.4–12.6	96.4	91.7–98.4	0.67	1.01
All tears	0.0	0.0–15.5	96.2	91.9–98.2	0.0	1.04
FT tears	0.0	0.0–32.4	96.5	92.5–98.4	0.0	1.04
Tendinosis	5.0	0.9–23.6	96.8	92.8–98.6	1.58	0.98
Q7						
All disease	51.2	36.5–65.8	65.0	56.7–72.5	1.46	0.75
All tears	57.1	36.6–75.5	63.7	55.9–70.8	1.57	0.67
FT tears	37.5	13.7–69.4	61.2	53.7–68.2	0.97	1.02
Tendinosis	45.0	25.8–65.8	62.0	54.3–69.2	1.19	0.89
Q8						
All disease	94.7	82.7–98.5	20.0	14.1–27.5	1.18	0.26
All tears	100.0	83.2–100	18.8	13.4–25.7	1.23	
FT tears	100.0	64.6–100	17.5	12.5–24.0	1.21	
Tendinosis	89.5	68.6–97.1	17.5	12.3–24.3	1.09	0.60
Superior posterior labral complex						
Q19						
All SLAP tears	37.7	25.9–51.2	69.1	60.5–76.6	1.22	1.89
Types II–V	54.2	35.1–72.1	70.4	62.7–77.1	1.83	0.65
Q20						
All SLAP tears	51.9	38.9–64.6	57.4	48.5–65.8	1.22	0.84
Types II–V	15.6	9.2–25.3	31.3	23.0–41.0	0.23	2.70
Q21						
All SLAP tears	59.3	46.0–71.3	32.8	25.1–41.5	0.88	1.24
Types II–V	75.0	55.1–88.0	36.8	29.6–44.8	1.19	0.68
Anterior instability						
Q22	70.0	56.3–80.9	68.5	60.0–75.9	2.22	0.44
Q23	76.0	62.6–85.7	81.3	73.6–87.1	4.05	0.30
Q29	30.0	19.1–43.8	94.5	89.1–97.3	5.44	0.74
Posterior instability						
Q22	54.6	28.0–78.7	58.4	50.8–65.7	1.31	0.78
Q23	36.4	15.2–64.6	66.5	58.9–73.2	1.08	0.96
Q24	63.6	35.4–84.8	58.3	50.6–65.6	1.53	0.62
Q25	72.7	43.4–90.3	41.6	34.3–49.2	1.25	0.66
Q26	63.6	35.4–84.8	76.7	69.6–82.5	2.73	0.47
Q29	27.3	9.8–56.6	88.6	82.8–92.6	2.38	0.82
Acromioclavicular joint arthritis						
Q30	87.0	75.6–93.6	12.1	7.3–19.2	0.99	1.07

Abbreviations: CI, confidence interval; FT, full thickness; LR, likelihood ratio; PT, partial thickness; SLAP, superior labrum anterior to posterior.

a All disease refers to any pathology affecting the supraspinatus tendon. This includes tendinosis, PT tears, and FT tears. All tears refer to both PT and FT tears.

The primary diagnoses following the physical examination agreed with the diagnoses made by the history in 74.6% of cases. Sixty-nine percent of these were correct according to the gold standard. The confidence change following physical examination was minimal on the VAS scale (2.69 ± 18.7). For those patients who the primary diagnosis after the history agreed with the diagnosis following the physical examination, only 10% did not correlate with the gold standard diagnosis. Seventeen percent of the primary and secondary diagnoses after the history were switched following the physical examination. Of these, 45% were identified correctly by the history, 23% by the physical examination, and the remaining were not identified by either the physical examination or history. The primary diagnosis changed entirely following the physical examination in 16.6% of cases. Of these, 47% were identified correctly with the history, 24% with the physical examination, and the remaining were not identified by either.

**Fig. 1:**
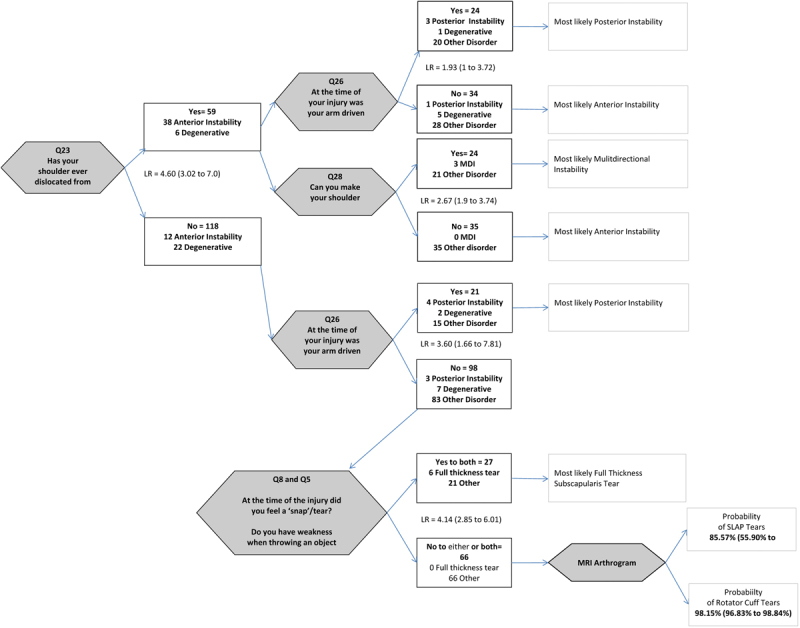
Diagnostic clinical decision algorithm using patient-reported history items for shoulder pathology.

The diagnostic decision algorithm is presented in Fig. 1 . Question 23 (“Has your shoulder ever dislocated from its socket?”) had the best combination of measurement properties and was therefore selected as the first question in the diagnostic algorithm. Of those who answered “yes” to this question, 38 had anterior labral tears, 6 had a degenerative labrum, and 15 had another disorder. Of those with another disorder, six had another type of instability (posterior, multidirectional, or atraumatic instability). Since these disorders could also present with shoulder dislocations, we assessed whether other questions could differentiate these diseases at this stage. Question 26 was found to have moderate diagnostic utility (LR = 1.93) for posterior instability, and Question 28 (“Can you make your shoulder come out?”) was able to differentiate multidirectional instability (LR = 2.67). For those patients who answered “no” to Question 23, analysis revealed that posterior instability could be predicted with Question 26 (LR = 3.60). Analysis with the remaining patients demonstrated that a combination of Questions 5 and 8 was diagnostic for full-thickness subscapularis tears (LR = 4.14). At this stage of the clinical decision algorithm, we found that the history items could not predict rotator cuff tears or SLAP lesions. We calculated a LR of an MRI arthrogram for rotator cuff tears 22 to be 86.7 and for SLAP lesions 21 to be 41. Using these LRs, we determined that the posttest probability of rotator cuff tear following an MRI arthrogram would be 98.15% (96.8–98.8%) and for SLAP lesions 83.67% (55.9–90%).

## Discussion

Diagnosis of shoulder pathology is one of the most challenging areas in orthopaedics as the clinical manifestations vary widely and pathologies often coexist. Our study demonstrates that the patient-reported history items for shoulder pathology are predictive of disease and can be useful in the diagnostic process. In particular, history items were good diagnostic indicators of anterior instability (Question 23), posterior instability (Question 26), and full-thickness subscapularis tears (Questions 5 and 8). History items for SLAP injuries and rotator cuff tears could not change the clinical impression of disease as their LRs were close to one. Physical examination changed the primary diagnosis made by the history in only 25% of cases, and of these, only 23% changed the diagnosis correctly, in 47%, the history was correct, and in the remaining cases neither the history nor physical examination was correct. We assessed whether MRI arthrogram could improve the ability to predict these disorders and found that the probability of disease could be improved to 83.7 and 93.2% for SLAP lesions and rotator cuff tears, respectively.

Several studies have established that a substantial portion of referrals to orthopaedic specialists are inappropriate. 9 23 24 25 Roland et al 25 found that 43% of referrals to their orthopaedic clinic could have been avoided. Similarly, Speed and Crisp 9 showed that only 42% of their referred sample was listed for a surgical intervention following orthopaedic consultation. Both concluded that referral guidelines might help make more efficient use of orthopaedic services and optimize patient care. A more efficient referral process could reduce the number of unsuitable patients being seen by the specialist and consequently reduce wait times and improve management of patients who require a specialist.

The use of triage systems to ensure referrals reach the most appropriate destination is a popular concept. This triage process begins with a referral sent by a primary care clinician and upon its arrival is directed by a gatekeeper. 9 Several pitfalls in the current system suggest a need for an improved triage system. First, general and primary care clinicians often have low levels of confidence in diagnosing and managing musculoskeletal disorders often referring patients when it is inappropriate or sending them for clinical tests that are not warranted. 25 26 In addition, this system is limited by the lack of information that is provided in the referral letter, and consequently, gatekeepers may have difficulty deciding where the referral should be sent to. We were able to construct a clinical decision algorithm that has the potential for implementation in the orthopaedic referral process. The algorithm is formatted as a decision tree whereby if a patient were to answer “no” to a question they would advance to the next, if they were to answer “yes” then the process would end and the patient would be referred to the appropriate management. If a patient were to get through the entire algorithm without responding “yes” to any question, we would recommend the patient be referred for a more invasive clinical test (MRI arthrogram) to assist in confirming a diagnosis before being referred to an orthopaedic specialist.

This algorithm has several advantages. First, only patients who answered “yes” to any item in the algorithm would be referred to an orthopaedic surgeon. This has the potential to reduce the number of unsuitable patients being seen by a specialist. In our study, if this algorithm was in place, the potential reduction in the number of patients seen by the surgeon would have been 37%. Second, this algorithm has the potential to reduce the number of costly or invasive tests that patients get referred for. Many patients who get referred to orthopaedic specialists have undergone at least one type of imaging modality, including X-ray, ultrasonography, and MRI. Our study found that patients do not have to undergo these examinations unless they proceed through the decision algorithm without a diagnosis. Using our algorithm, only 37% of patients would have been referred for an MRI arthrogram. Primary care clinicians need to be informed that musculoskeletal patients do not need to be sent for these modalities as part of their work-up prior to referral. This has the potential to reduce the cost to health care resources, as only a fraction of musculoskeletal referrals will be sent for costly examinations. Third, as a health care specialist is not needed to collect the data required for our algorithm, this system may lend itself to electronic administration. In an era of ever-advancing technology, paperless charting, electronic access to patient care guidelines, and computerized decision tools promise to improve patient care. Electronic methods of triaging have been assessed in an emergency department setting and were found to improve allocation of patients compared with traditional triaging methods. 27 Future research efforts could assess whether such an instrument can be utilized in the referral process electronically in this orthopaedic population.

Although this decision tool has the potential to improve the efficiency of orthopaedic services, it is necessary to validate this tool in the orthopaedic shoulder population. Future research should focus on determining if this triage system can successfully allocate patients. This research would inform us whether this tool is useful in a clinical setting.

### Limitations

A limitation of our study is that patients enrolled in our study were referred to a tertiary care orthopaedic clinic; therefore, the results should be generalized to only those types of patients. Although the generalizability is limited, the strengths of this study include its large sample size, which enable us to provide precise measures of the specificity, sensitivity, and LRs of the history items. In addition, this study involves four surgeons in two different cities in Ontario, Canada, which increases the applicability of the results. Consequently, there is enormous potential for knowledge transfer, in that our results will be used to guide practice, teach medical students, residents, and fellows, and will create a more research friendly atmosphere.

## Conclusion

Based on these study results, we found that the patient-reported history is able to diagnose anterior instability, posterior instability, and subscapularis tears. In fact, the physical examination and history agreed in 75% of cases. Of those that did not agree, the physical examination misdirected the diagnosis in 47% of our cases. We can conclude that these have the potential to assist in the triage process. In addition, patients should not be sent for diagnostic imaging without first triaging; moreover, if the patient gets to the end of the decision tree without a diagnosis, MRI arthrogram is an appropriate imaging modality to distinguish both rotator cuff tears and SLAP lesions.
